# Cosmetic Reconstruction of Frontotemporal Depression Using Polyethylene Implant after Pterional Craniotomy

**DOI:** 10.1155/2018/1982726

**Published:** 2018-10-21

**Authors:** Sang Hyuk Im, Jongkeun Song, Sang Kyu Park, Eun Young Rha, Young-Min Han

**Affiliations:** ^1^Department of Neurosurgery, College of Medicine, The Catholic University of Korea, Seoul, Republic of Korea; ^2^Department of Plastic and Reconstructive Surgery, College of Medicine, The Catholic University of Korea, Seoul, Republic of Korea; ^3^Department of Neurosurgery, Naeun Hospital, Incheon, Republic of Korea

## Abstract

**Purpose:**

Pterional craniotomy is a useful approach for the treatment of a variety of intracranial pathologies. However, it can result in temporal hollowing, which causes significant craniomaxillofacial asymmetry and esthetic deformity. The present study was performed to determine the postoperative outcomes of patients following frontotemporal depression reconstruction using a high-density porous polyethylene (HDPE) implant (Medpor®; Stryker, Kalamazoo, MI) after pterional craniotomy.

**Materials and Methods:**

The patients had undergone reconstruction of frontotemporal depression using Medpor® implants after pterional craniotomy at our medical institution during the period from February 2010 to March 2014. We evaluated the thickness and volume of both the temporalis muscle and Medpor® implant through a retrospective review of the medical records and computed tomography (CT) scans of 92 patients.

**Results:**

The mean temporalis muscle thickness ratio (muscle thickness of the affected side/nonaffected side) was 0.61 ± 0.16. The mean reconstructed temporalis muscle thickness ratio (muscle and Medpor® implant thickness of affected side/muscle thickness of nonaffected side) was 1.15 ± 0.02. The mean temporalis muscle volume ratio (muscle volume of affected side/nonaffected side) was 0.67 ± 0.02. The mean reconstructed temporalis muscle volume ratio (muscle and Medpor® implant volume of affected side/muscle volume of nonaffected side) was 1.18 ± 0.02.

**Conclusions:**

Temporalis muscle thickness and volume were significantly decreased on the affected side after pterional craniotomy. Reconstruction of frontotemporal lesions using Medpor® implants after the pterional approach improved temporal hollowing without additional complications.

## 1. Introduction

Pterional craniotomy, first described by Yasargil in 1975, is one of the most commonly used techniques in vascular neurosurgery [1–3]. Although this approach enables easy access to a variety of lesions in the anterior and middle cranial fossae, superior aspect of the posterior cranial fossa, sellar and parasellar regions, superior orbital fissure, and cavernous sinus [1, 4–7], it has several limitations, such as temporalis muscle atrophy and facial nerve damage, which are matters of great concern to patients even with successful postoperative outcome. Temporal hollowing, that is, contour irregularity in the frontotemporal region, develops due to disruption of the ligamentous attachments among soft tissue and compromised blood supply to the temporoparietal fascia and superficial temporal fascia during dissection of the frontotemporal area [8–12]. This can cause significant esthetic deformity and eventually lead to a decrease in patient compliance in relevant cases.

A number of modified dissection techniques using a pterional approach have been developed to prevent temporal fossa depression and reduce the risk of frontal branch injury. However, there has been debate regarding the effectiveness of methods to achieve both excellent clinical (by ensuring a sufficient visual field) and cosmetic results [13–15]. Moreover, although various autograft and allograft materials have been described for use in reconstruction of temporal hollowing [16–20], there have been few studies assessing the cosmetic effects based on objective measurements.

Here, we present our technique involving frontotemporal reconstruction using a high-density porous polyethylene (HDPE) implant (Medpor®; Stryker, Kalamazoo, MI) following pterional craniotomy and evaluate the outcomes of temporal augmentation.

## 2. Materials and Methods

### 2.1. Study Population

In this single-center, retrospective study, we evaluated the medical records and computed tomography (CT) scans of 99 patients who had undergone temporal hollowing reconstruction using Medpor® temporal implants after pterional craniotomy at Incheon St. Mary's Hospital between February 2010 and March 2014.

Inclusion criteria for the present study were as follows:

(1) Patients diagnosed with unruptured aneurysms without neurological deficits

(2) Patients who underwent pterional craniotomy

(3) Patients who underwent frontotemporal reconstruction with a Medpor® temporal implant

(4) Patients who underwent postoperative evaluation, including clinical outcome, esthetic outcome, and radiological examination, at 3 months postoperatively

Exclusion criteria for the present study were as follows:

(1) Diagnosis of with ruptured aneurysm

(2) Neurological deficits

(3) Bilateral pterional craniotomy

(4) Procedures affecting the thickness of soft tissue of the temporal area on the affected or nonaffected side

(5) No postoperative evaluation (because the patient did not visit the outpatient clinic of the Department of Neurosurgery of our medical institution at 3 months postoperatively)

The present study was approved by the Institutional Review Board (IRB) of our medical institution (IRB approval number: OC17RESI0159). The informed consent requirement was waived due to the retrospective nature of the study. All data was analyzed anonymously and according to the principles in the Declaration of Helsinki (1975, revised in 2008).

A total of 92 patients were included in this study and we evaluated the clinical outcome, degree of esthetic satisfaction, and radiologic data in these cases.

### 2.2. Surgical Technique

**Figure 1:**
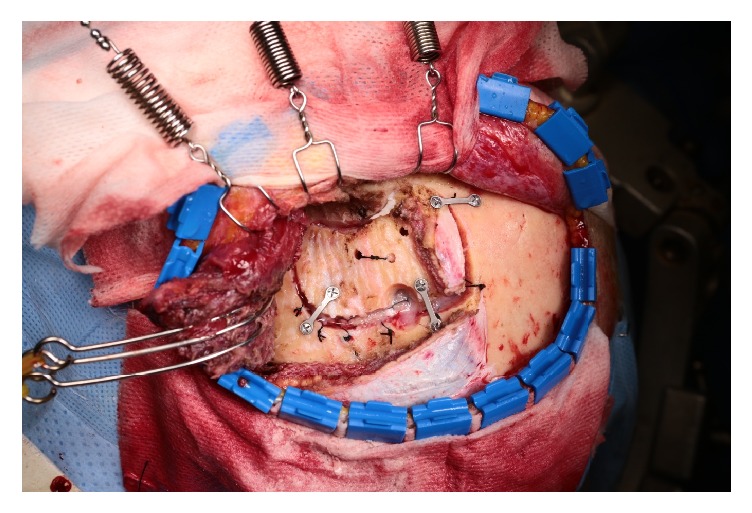
Intraoperative photograph after pterional craniotomy. The bone flap was secured with metallic plates and screws under the elevated temporalis muscle.

**Figure 2:**
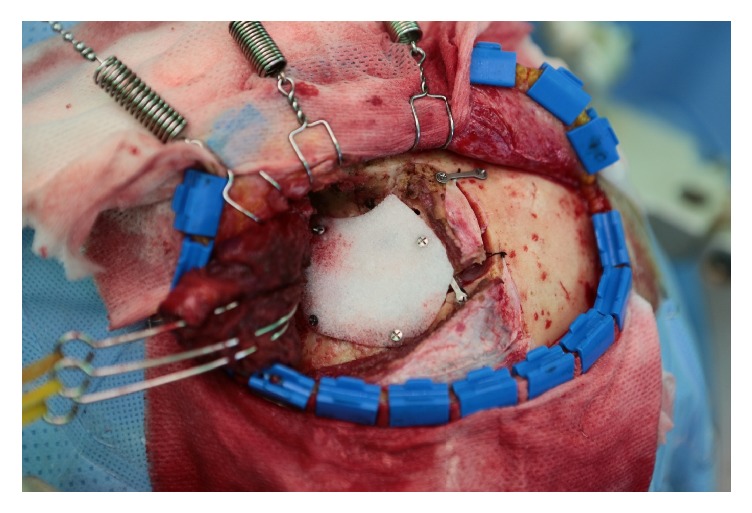
Intraoperative photograph after Medpor® implant insertion. The Medpor® implant was applied to the secured bone flap and fixed with metallic screws.

**Figure 3:**
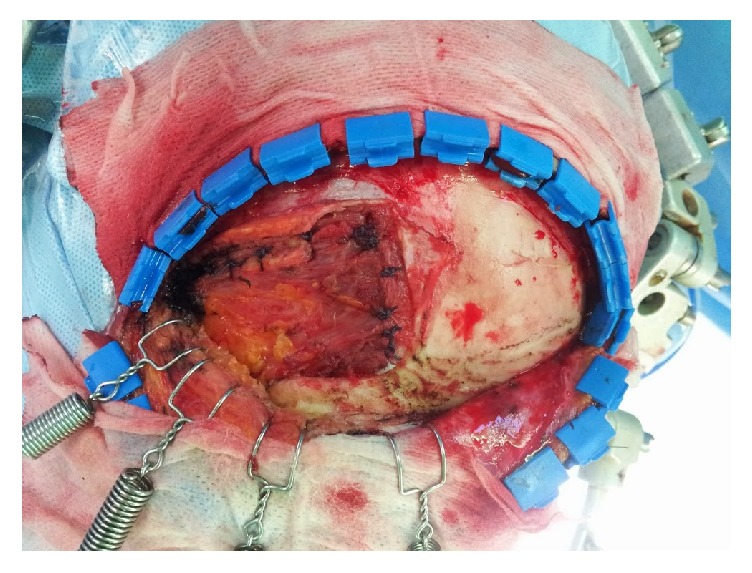
Intraoperative photograph after suspension of temporalis muscle. After application of the Medpor® implant to the bone flap, the elevated temporalis muscle was approximated to the cutting end of the temporalis muscle attached to the bone.

After completion of the cranial operation using the pterional approach, which was performed as described by Spetzler and Lee [21], the bone flap was secured to the skull with metallic plates and screws (Figure 1). The prefabricated high-density polyethylene (HDPE) implant (Medpor; Stryker, Kalamazoo, MI) was tailored with heavy scissors to fit the dimensions and contours of the defect accurately. Once positioned correctly, the Medpor® implant was secured in place with metallic screws (Figure 2). The temporalis muscle was then suspended and the soft tissue was approximated (Figure 3).

### 2.3. Quantitative Measurement of the Thickness and Volume of Temporalis Muscle

**Figure 4:**
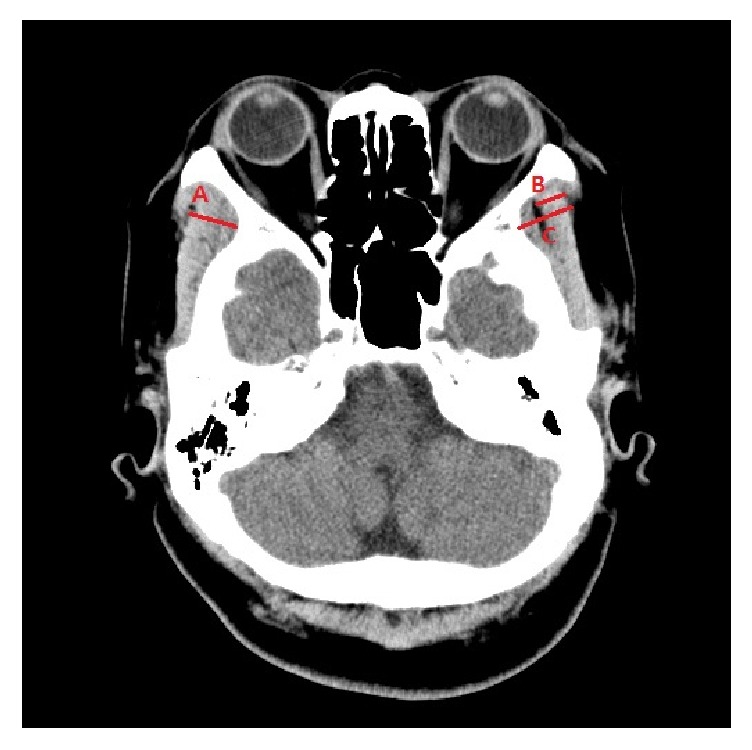
Thickness of the temporalis muscle and Medpor® implant. (A) Temporalis muscle thickness of the nonaffected side. (B) Temporalis muscle thickness of the affected side. (C) Temporalis muscle thickness of the nonaffected side with Medpor®.

**Figure 5:**
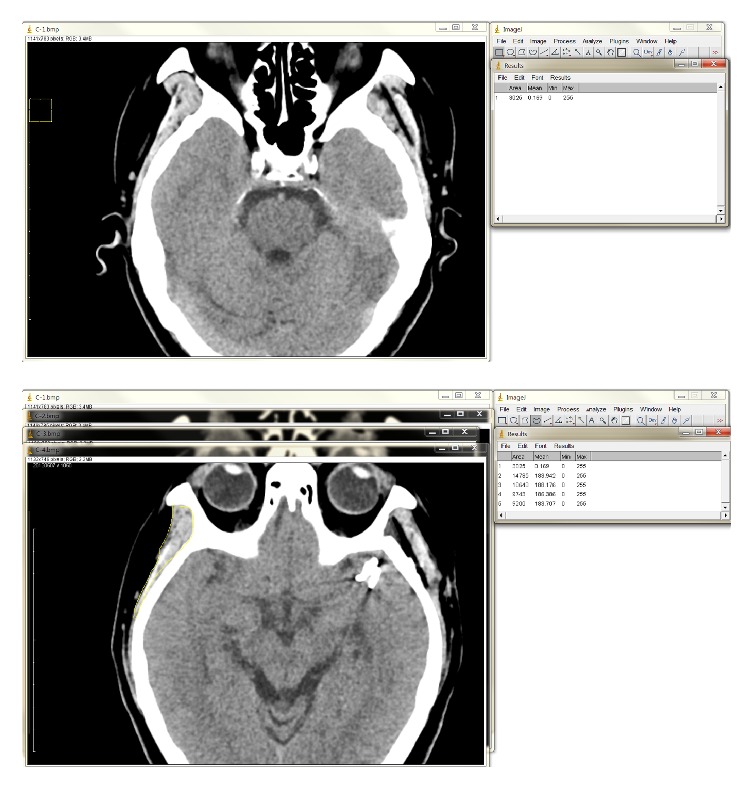
Calculation of temporalis muscle volume using ImageJ. (Top) A square with the length of the reduced scale (1cm) in an axial section of a computed tomography (CT) image was drawn. The number of pixels inside the square per unit area (1 cm^2^) was calculated to be 3025 using the rectangular selection tool. (Bottom) A curved line was drawn along the margin of the temporalis muscle using the freehand selection tool. The number of pixels inside the region of interest was determined.

The thicknesses of the temporalis muscle and Medpor® implant were measured on brain CT axial images. The perpendicular line from the sphenoid greater wing to the outer margin of the temporalis muscle was defined as the thickness of the temporalis muscle. The thickness of the temporalis muscle reconstructed with a Medpor® implant was measured in the same manner from the sphenoid greater wing to the outer margin of the temporalis muscle, including the Medpor® implant (Figure 4). The volumes of the temporalis muscle and Medpor® implant were measured as described previously [22], using the public domain, Java-based image processing software package ImageJ, developed at the National Institutes of Health (NIH, Bethesda, MD) (Figure 5) [23].

### 2.4. Esthetic Outcomes

Esthetic results, from both the surgeon's and the patient's perspectives, were analyzed and classified as excellent, good, regular, or poor using a predetermined scale.

### 2.5. Statistical Analysis

Statistical analyses were performed using SPSS for Windows software (ver. 18.0; SPSS Inc., Chicago, IL). All data are expressed as the average ± standard deviation (SD). The paired* t*-test was used to compare the ratios of thickness and volume between the affected and nonaffected sides. In all analyses,* P* < 0.05 was taken to indicate statistical significance.

## 3. Results 

**Figure 6:**
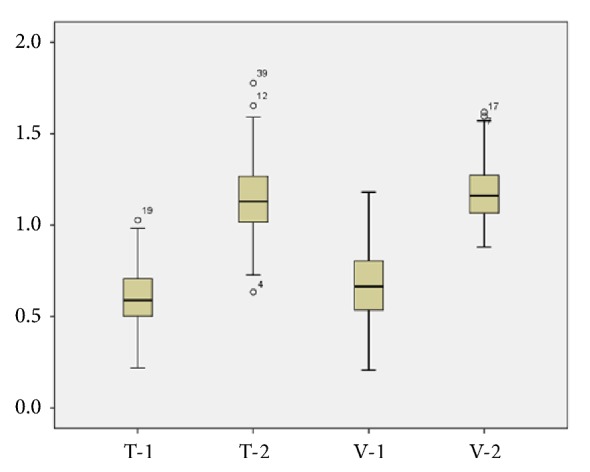
Box plot showing the ratio between the thickness and volume of the affected and nonaffected temporalis muscle. T-1, temporalis muscle thickness ratio (0.61 ± 0.16); T-2, reconstructed temporalis muscle thickness ratio (1.15 ± 0.02); V-1, temporalis muscle volume ratio (0.67 ± 0.02); V-2, reconstructed temporalis muscle volume ratio (1.18 ± 0.02),* P* ≤ 0.001.

The mean ratio of temporalis muscle thickness between the affected and nonaffected sides was 0.61 ± 0.16. The mean ratio of reconstructed temporalis muscle and Medpor® thickness between the affected and nonaffected sides was 1.15 ± 0.02. The mean ratio of temporalis muscle volume between the affected and nonaffected sides was 0.67 ± 0.02. The mean ratio of reconstructed temporalis muscle and Medpor® volume between the affected and nonaffected sides was 1.18 ± 0.02. The thickness and volume of the temporalis muscle were significantly increased after reconstruction with the Medpor® implant (both,* P* ≤ 0.001) (Table 1 and Figure 6). Overall patient satisfaction (excellent and good) was observed in 83 of 92 patients (90.2%), and there were no additional complications.

## 4. Discussion

Temporal hollowing is a contour irregularity in the frontotemporal area, which commonly develops following surgical dissection in the temporal region, including via the intracranial access procedure [24, 25]. Temporal hollowing can cause significant craniomaxillofacial asymmetry, esthetic deformity, and serious cosmetic concern in patients, even when there is an excellent postoperative functional outcome. The proposed mechanisms of temporal hollowing include devascularization, denervation, or disruption of the fat pads or temporalis muscle [8, 24, 26]. In pterional craniotomy using the “interfascial temporalis flap” technique, the intermediate temporal fat pad is split vertically to create two separate composite flaps. The anteriorly reflected flap includes skin, superficial temporal fascia, partial superficial layer of the deep temporal fascia, partial intermediate temporal fat pad, and partial deep layer of the deep temporal fascia. The posteroinferiorly reflected flap includes the temporalis muscle, deep temporal fat pad, partial deep layer of the deep temporal fascia, and partial intermediate fat pad [25]. Use of the myocutaneous flap technique has been reported to avoid fat pad dissection. De Andrade et al. [27] reported that a myocutaneous flap group showed significantly lower levels of temporal hollowing compared to an interfascial temporalis group. However, this technique limits exposure of the anteroinferior temporal fossa.

Various therapeutic methods have been developed for reconstruction of temporal hollowing using both autograft and alloplast materials. Autologous bone has the advantages of being genetically identical to the patient, possessing the potential for growth and replacement of host cells, and a low incidence of infection [16, 28–32]. However, it causes donor site morbidity, graft resorption, and difficulties with contouring, and additional time is required for harvesting of the graft [31, 33]. Moreover, this technique cannot adequately restore deficient soft tissue [24]. Autologous fat grafting can also be used for correction of volume loss of the temporal lesion [34–37]. It is characterized by minimal donor site morbidity and minimal postoperative pain, but isolated use of a fat graft is inadequate for reconstruction of temporal depression because of the variable degree of graft absorption and the subsequent need for serial grafting. Adjuvant techniques combined with alloplastic grafts would be effective for temporal hollowing [24].

Alloplastic materials have emerged for replacement of autologous tissue; these are biocompatible with the surrounding bone and soft tissue, durable over time, easily molded, and associated with low donor site morbidity [33, 38]. Methyl methacrylate, an acrylic-based resin, is biocompatible, inelastic, strong, and readily available [33]. However, it is an inert material and injures local tissues due to the release of heat during shaping of the material. There is also an elevated risk of infection when it is applied to contaminated areas, such as the paranasal sinuses, and in cases of prior infection [39]. Hydroxyapatite, the primary mineral component of bone, has excellent tissue compatibility and a high capacity for osteoconduction and osteointegration. However, it has a high rate of infection and cannot bear significant loads [33, 39].

Porous polyethylene is commonly used for facial augmentation or reconstruction of various defects in the facial skeleton. Polyethylene is composed of straight-chain aliphatic hydrocarbons, is inert, and causes little tissue reactivity [40]. The vascular and tissue ingrowth of HDPE caused by the porous nature of the materials contributes to long-term stability and resistance to infection. There is also little evidence of implant degradation or resorption [29, 40].

Liu et al. [29] reported a surgical technique using Medpor® porous polyethylene implants in 611 standard cranial and skull base procedures, including treatment for temporal hollowing after pterional craniotomy. They noted satisfactory compensation of temporalis muscle atrophy and no implant-related complications. Rapidis et al. [41] discussed the use of a prefabricated porous HDPE temporal implant after temporalis myofascial flap (TMF) transposition in a retrospective review of 21 patients. Long-term functional and esthetic results were shown to be stable even in patients receiving postoperative radiotherapy. Mericli et al. [24] presented four patients treated for temporal hollowing correction using HDPE after either craniotomy or extirpative surgery for neoplasm. They also described subtypes of temporal defects treated by appropriate reconstruction methods and reported that HDPE was a safe, well-studied, and easily handled biomaterial for recovery of temporal hollowing.

As outlined above, there have been several reports regarding reconstruction techniques using Medpor® implants following the pterional approach. However, there have been few studies regarding the severity of temporal hollowing or the effects of reconstruction using Medpor® implants based on objective measurements.

This study demonstrated the extent of temporal hollowing after pterional craniotomy, as well as the postoperative outcomes of frontotemporal reconstructions done using Medpor® implants, based on the objective values in 92 patients. The temporalis muscle was atrophied to about 39% and 33% in thickness and volume, respectively, after application of the pterional approach. The temporal hollowing was significantly ameliorated after reconstruction using Medpor® implants, with increases of about 15% and 18% in thickness and volume, respectively, compared to the nonaffected side. Overreconstruction of the affected side may be due to postoperative changes, such as inflammation and scarring between the injured periosteum and Medpor® implant or between the Medpor® implant and temporalis muscle layer.

This study had several limitations. First, it was retrospective in design. Second, it compared the thickness and volume of temporalis muscle between the affected and nonaffected sides but did not consider the possibility of temporalis muscle asymmetry between the two sides. Third, only deep soft tissue including the temporalis muscle was compared. The superficial soft tissue layer outside the temporalis muscle was not included in the measurement of temporal hollowing because this tissue could be influenced by multiple factors, such as inflammation, swelling, and scarring. Further studies to determine the soft tissue factors affecting temporal hollowing are required.

## 5. Conclusions

Significant temporalis muscle atrophy affected postoperative temporal hollowing following pterional craniotomy. Reconstruction of frontotemporal lesions using Medpor® temporal implants after the pterional approach effectively improved temporal hollowing without additional complications.

## Figures and Tables

**Table 1:** Baseline characteristics of temporal lesions.

	Average ± SD	P-value
Temporalis muscle thickness ratio	0.61±0.16	≤ 0.001
Reconstructed temporalis muscle thickness ratio	1.15±0.02	
Temporalis muscle volume ratio	0.67±0.02	≤ 0.001
Reconstructed temporalis muscle volume ratio	1.18±0.02	

## Data Availability

The [supplemental] data used to support the findings of this study are included within the supplementary information file.

## Supplementary Materials

Supplementary Materials S1 Table. The ratio of temporalis muscle thickness and volume of 92 patients.
