# Supplementary material for: Cosmetic Reconstruction of Frontotemporal Depression Using Polyethylene Implant after Pterional Craniotomy
Source: Biomed Res Int. 2018 Oct 21;2018:1982726. doi: 10.1155/2018/1982726 (PMC6215591; doi:10.1155/2018/1982726)
Supplement: Supplementary Materials — S1 Table. The ratio of temporalis muscle thickness and volume of 92 patients. [file 1982726.f1.zip › 1982726.f1/Pages from 1982726.v2.pdf]

Supplementary Materials

S1 Table. The ratio of temporalis muscle thickness and volume of 92 patients

Number	T-1	T-2	V-1	V-2
1	0.49	1.26	0.77	1.09
2	0.39	0.73	0.88	1.31
3	0.77	1.40	1.18	1.24
4	0.29	0.63	0.54	1.07
5	0.40	1.05	0.73	1.13
6	0.64	1.20	0.69	1.24
7	0.22	0.86	0.78	1.62
8	0.45	1.06	0.53	1.37
9	0.44	0.86	0.62	0.88
10	0.65	1.36	0.95	1.52
11	0.60	1.05	0.50	1.10
12	0.40	1.65	0.48	1.11
13	0.54	1.40	0.63	1.05
14	0.25	1.20	0.29	1.02
15	0.50	1.07	0.90	1.52
16	0.77	1.48	0.81	1.46
17	0.55	1.53	0.93	1.60
18	0.66	1.12	0.88	1.15
19	1.03	1.55	0.51	1.02
20	0.27	1.36	0.41	1.39
21	0.58	1.19	0.88	1.35
22	0.50	0.96	0.71	1.13
23	0.50	1.41	0.63	1.22
24	0.68	1.11	0.71	1.27
25	0.46	1.19	0.55	1.40
26	0.76	1.45	0.83	1.40
27	0.62	1.25	0.84	1.13
28	0.50	0.95	0.45	0.92
29	0.65	1.07	0.91	1.17
30	0.55	0.94	0.57	0.97

31	0.54	1.59	0.60	1.49
32	0.67	1.37	0.71	1.28
33	0.56	0.84	0.64	1.28
34	0.54	1.19	0.51	1.21
35	0.67	1.34	0.84	1.20
36	0.52	1.11	0.67	1.11
37	0.67	1.08	0.21	1.35
38	0.71	1.16	0.46	1.01
39	0.98	1.78	0.49	1.10
40	0.81	1.41	0.81	1.11
41	0.70	1.16	0.77	1.17
42	0.59	1.02	0.78	1.19
43	0.86	1.38	0.53	1.08
44	0.55	1.04	0.43	1.03
45	0.89	1.55	0.54	1.33
46	0.73	1.15	0.71	0.98
47	0.55	1.04	0.51	1.22
48	0.45	0.89	0.49	1.16
49	0.50	1.14	0.48	1.18
50	0.62	0.94	0.56	1.13
51	0.65	0.93	0.71	1.10
52	0.44	1.14	0.53	1.07
53	0.53	0.83	0.56	1.05
54	0.64	0.99	0.57	0.99
55	0.67	1.13	0.57	1.26
56	0.73	1.15	0.56	1.20
57	0.69	1.06	0.52	1.07
58	0.77	1.14	0.47	1.18
59	0.59	1.11	0.66	1.05
60	0.46	0.96	0.65	0.97
61	0.48	0.88	0.66	1.01
62	0.84	1.22	0.87	1.38
63	0.54	0.86	0.80	1.04

64	0.61	1.27	0.76	1.19
65	0.76	1.14	0.80	1.07
66	0.79	1.07	0.41	1.09
67	0.81	1.21	0.88	1.06
68	0.56	0.94	0.72	1.19
69	0.78	1.08	0.57	0.95
70	0.97	1.33	0.72	1.20
71	0.68	1.16	0.77	1.22
72	0.54	1.01	0.64	1.04
73	0.51	0.98	0.78	1.09
74	0.81	1.22	0.68	1.18
75	0.53	0.98	0.67	1.16
76	0.71	1.13	0.84	1.28
77	0.56	0.82	0.62	0.99
78	0.46	0.89	0.74	1.05
79	0.71	1.09	0.65	1.24
80	0.73	1.29	0.98	1.34
81	0.55	1.03	0.81	1.14
82	0.49	0.87	0.76	0.91
83	0.57	1.04	0.85	1.44
84	0.66	1.11	0.95	1.17
85	0.74	1.13	1.08	1.22
86	0.38	1.40	0.65	1.34
87	0.57	1.21	0.61	1.08
88	0.86	1.27	0.84	1.34
89	0.72	1.19	1.14	1.13
90	0.53	1.36	0.29	1.57
91	0.47	1.11	0.30	1.01
92	0.70	1.17	0.35	1.06

T-1, temporalis muscle thickness ratio; T-2, reconstructed temporalis muscle thickness ratio; V-1, temporalis muscle volume ratio; V-2, reconstructed temporalis muscle volume ratio
